# Atypical Human Effector/Memory CD4^+^ T Cells With a Naive-Like Phenotype

**DOI:** 10.3389/fimmu.2018.02832

**Published:** 2018-12-03

**Authors:** Nadia Caccamo, Simone A. Joosten, Tom H. M. Ottenhoff, Francesco Dieli

**Affiliations:** ^1^Central Laboratory of Advanced Diagnosis and Biomedical Research (CLADIBIOR), University of Palermo, Palermo, Italy; ^2^Department of Biopathology and Medical Biotechnologies, University of Palermo, Palermo, Italy; ^3^Department of Infectious Diseases, Leiden University Medical Center, Leiden, Netherlands

**Keywords:** CD4^+^ T cells, naive T cells, effector T cells, immunological memory, cytokines, infection, *M. tuberculosis* infection

## Abstract

The induction of adaptive immunological memory, mediated by T and B cells, plays an important role in protective immunity to pathogens induced by previous infections or vaccination. Naive CD4^+^ T cells that have been primed by antigen develop into memory or effector cells, which may be distinguished by their capability to exert a long-term and rapid response upon re-challenge by antigen, to produce distinct cytokines and surface marker expression phenotypes such as CD45RA/RO, CD27, CD62L, and CCR7. Moreover, a distinct lineage of memory T cells populates tissues (tissue-resident memory T cells or T_RM_ cells) which orchestratea the response to pathogens re encountered at tissue sites. Recent evidence, however, has highlighted that CD4^+^ naive T cells are much more heterogeneous that previously thought, and that they harbor diversity in phenotypes, differentiation stages, persistence, functions, and anatomic localizations. These cells represent cellular subsets that are extremely heterogeneous and multifunctional at their very initial stages of differentiation, with the potential to become “atypical” memory and effector cells. In this mini review, we focus on recently obtained data from studies in humans, in which this newly recognized heterogeneity in the naive T cell pool was discovered in terms of surface marker expression, cytokine production, or transcriptomic profiles. The deep analysis of immune functions at the single cell level combined with a better understanding of the generation and maintenance of the various atypical memory CD4^+^ T cell subsets with a naive-like phenotype will be important in immune-monitoring of vaccination and immunotherapies in infectious diseases.

## Introduction

CD4^+^ T lymphocytes mature in the thymus after passing through the processes of positive and negative selection and migrate to secondary lymphoid organs. These mature T lymphocytes, that have not yet encountered antigen (naive T cells), continuously recirculate between secondary lymphoid organs and blood. Upon recognition of specific antigen/MHC complexes naive CD4^+^ T cells proliferate and differentiate toward effector T cells, which provide immediate protection. Most of these effector T cells subsequently die by apoptosis, but a subset of antigen-specific T cells will persist in an individual as memory T cells (1). There are two types of memory T cells in the circulation, central (T_CM_) and effector (T_EM_) memory T cells: the former show self-renewal potential, home to secondary lymphoid organs but lack effector functions, while the latter possess immediate effector functions and can rapidly migrate to peripheral tissues to provide antigen elimination (2). Moreover, a distinct lineage of tissue-resident memory T cells (T_RM_ cells) has been described in the last years, which are confined to different tissues and orchestrate the response to pathogens re encountered at tissue sites.

Due to thymic regression with age, the survival of the naive T cell pool is maintained by homeostatic mechanisms in the periphery, including IL-7 and low affinity T-cell receptor (TCR)-recognized self peptide/MHC complexes, which however do not induce differentiation into central or effector memory T cells (2). Since naive CD4^+^ T cells in humans have a lifespan of 6–10 years (3), this homeostatic mechanism maintains a broad repertoire of T cell subsets and TCR specificities in the periphery over prolonged periods of time.

**Figure 1:**
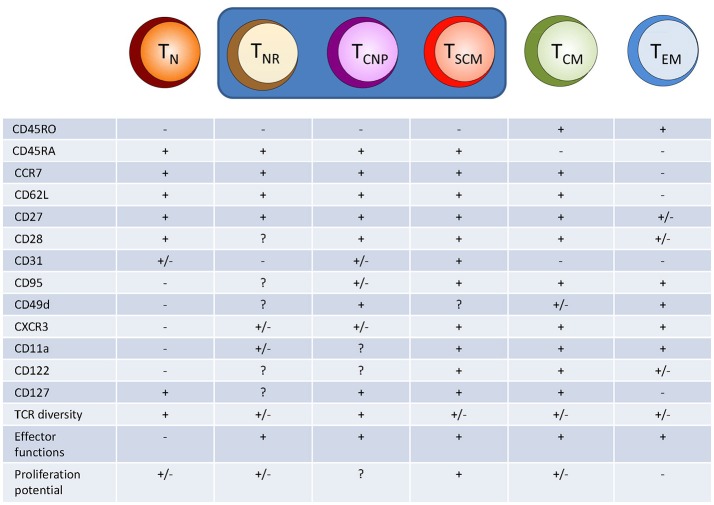
Hypothetical model of human CD4^+^ T cell differentiation. Naive T cells (T_N_) upon specific antigen stimulation progressively differentiate into different population of effector/memory cells, including T cells with a naive-like phenotype but exerting several different effector functions, such as cytokine production (T_NR_, T_CNP_, and T_SCM_ cells). T_NR_, naive receptor memory T cells, T_SCM_, stem memory T cells; T_CM_, central memory T cells; T_EM_, effector memory T cells.

The naive CD4^+^ T cell compartment has long been considered as consisting of a homogeneous population of antigen-inexperienced cells (2), identified by specific surface markers. In humans, naive CD4^+^ T cells typically express CCR7, CD62L, and CD45RA, while lacking expression of CD45RO (2). CCR7 and CD62L are involved in the homing of T cells to secondary lymphoid organs (SLOs) and interact with ligands expressed on high endothelial venules (HEV). CD45RA and CD45RO play a role in TCR signal transduction, and their expression characterize the different T cells subsets (4). However, there is increasing evidence that this phenotypic identification of naive T cells includes populations equipped with memory and/or effector functions, thus making it clear that the “naïve” CD4^+^ T cell compartment spans a whole spectrum of cells with different properties (Figure 1).

Here we will review specifically the recent evidence for the existence of distinct subsets of CD4^+^ effector/memory T cells with a naive phenotype, as they may play an important role in different clinical settings, and need to be considered in immune-monitoring strategies in vaccination and immunotherapy. Similar subsets of CD8^+^ effector/memory T cells with a naive phenotype have also been described, but will not be discussed further here as they have been described elsewhere (4).

## Effector/Memory CD4^+^ T cells with a naive phenotype

### Naive receptor^+^ CD4^+^ T cells (T_NR_^+^)

Within the human naive CD4^+^ subset, classically identified as CD45RO^−^, CD62L^+^, CD27^+^, and CD11a^dim^, Song et al. (5) have identified in the blood and tonsils of healthy individuals, a population of cells expressing CCR4 and/or CXCR3, which accounted for ~20% of all cells in the naive compartment. They designated this CD4^+^ population as an alternative naive phenotype, T${}_{\text{NR}}^{+}$ (T naive receptor^+^) cells. The T${}_{\text{NR}}^{+}$ cells have T cell receptor (TCR) rearrangement excision circles (TREC) numbers intermediate between naive and memory CD4^+^ T cells, while having lost CD31 expression, a marker that usually has been proposed as identifier of naive cells that have undergone homeostatic division. In fact, the number of TRECs diminishes in each cell division (since TRECs are not copied during cell division) and CD31 is expressed on naive CD4^+^ T cells that have recently egressed from the thymus where its expression is downregulated following TCR stimulation. Moreover, T${}_{\text{NR}}^{+}$ cells use a broad TCR Vβ repertoire but with some proof of clonal expansion, suggesting antigen specificity, and upon polyclonal stimulation with PMA and ionomycin they produced low, yet significant levels of either IL-4 or IFN-γ, characteristic of differentiated cells. However, it is not known whether T${}_{\text{NR}}^{+}$ cells produce single or multiple cytokines, including TNF-α. Therefore, although the CD4^+^ T${}_{\text{NR}}^{+}$ cells express a naive surface phenotype, they display typical features of a memory cell population.

### Stem cell memory CD4^+^ T cells (T_SCM_)

Within the CD4^+^ (and CD8^+^) T cell compartment of CD45RA^+^, CCR7^+^ phenotypically naive cells, a proportion of T cells is clearly distinct from classically naive T cells as they display phenotypical and functional characteristics reminiscent of effector and memory T cells. Among these naive-like memory T cells, a subset termed stem cell memory T (T_SCM_) cells has been identified based on the expression of CD95, CD122, CXCR3, and the integrin CD11a (6, 7). CD95 is a surface marker molecule belonging to members of the TNF family and functions as a death receptor because its signaling induces apoptosis through activation of caspase-8 (8). Recent data, however, have highlighted that CD95 may play a role as a costimulatory surface molecule of CD4^+^ and CD8^+^ T cells (9, 10). This molecule is upregulated in activated T cells and stably expressed in all memory cells (11). CD122 is the β-chain of the IL-2 and IL-15 receptors. It is progressively upregulated along the differentiation of memory T cells, displaying different functions in terms of expansion, survival, and homeostatic features of memory T cell subsets (12, 13). The chemokine receptor CXCR3 (6) regulates lymphocyte trafficking, possibly allowing their migration into inflammatory sites guided by CXCR3 ligands, CXCL9 (MIG), CXCL10 (IP-10), and CXCL11 (I-TAC) (14). Finally, CD11a (the α subunit of LFA1) is involved in homing to SLOs and inflamed sites and in the adhesion to antigen-presenting cells (6).

These so-called T_SCM_ cells are generated during primary immune responses and are considered a reservoir for the memory pool, in line with their unique ability for self-renewal through still unidentified mechanisms (6). T_SCM_ cells express a substantially lower TREC content than the naive T cell population as a whole, similar to non-naive T cells, and can rapidly acquire effector functions (cytokine production) upon antigenic and homeostatic stimulation. Despite low TREC levels, high expression levels of the intracellular proliferation marker Ki-67 and a restricted TCR repertoire (15) (characteristics suggesting that they have undergone several rounds of division), T_SCM_ cells preserve their naive-like phenotype. It has been suggested that CD8^+^ T_SCM_ cells induced by a yellow fever vaccine in humans can stably persist for more than 25 years and are maintained by extensive proliferation (16).

Interestingly, Mpande et al. (17) have also reported the identification of seemingly naive CD4^+^ T cells that are induced by primary *M. tuberculosis* infection. These cells are distinct from naive T cells, display a T_SCM_ phenotype and produce IL-2, TNF-α, and IFN-γ. This has been identified by transcriptomic analysis, showing that bulk CD4^+^ T_SCM_ and *M. tuberculosis*-specific T_SCM_ cells expressed chemokine receptor and cytotoxic molecule transcripts, which were mostly undetectable in bulk T naive cells. Moreover, the comparison of the different subsets of CD4^+^ T cells showed that *M. tuberculosis*-specific T_SCM_ cells possessed the least differentiated *M. tuberculosis*-specific phenotypic and functional profile, suggesting that *M. tuberculosis*-specific T_CM_ cells appear as an intermediate subset before T_SCM_ cells further differentiate into *M. tuberculosis*-specific effector CD4^+^ T cells.

### Cytokine-producing naive CD4^+^ T cells (T_CNP_)

Very recently, we have identified in the peripheral blood of patients with tuberculosis a novel human effector/memory CD4^+^ T cell subset with a non-classical, naive-like T cell phenotype. These cells were CD45RO^−^, CD45RA^+^, CCR7^+^, CD62L^+^, CD27^+^, and capable of rapidly secreting multiple cytokines (IFN-γ, TNF-α, IL-2) in response to different *M. tuberculosis* antigens (18). We have designated this CD4^+^T cell population as T_CNP_ cells (T cells that are able to produce cytokines with a naive phenotype). T_CNP_ cells were further phenotyped as CD95^lo^ CD28^int^ CD49d^hi^ CXCR3^hi^ and a sizeable fraction (ranging from 50 to 80%) also expressed CD31. Following curative tuberculosis treatment, the size of this T cell subset significantly decreased, suggesting that these cells are markers of active tuberculosis disease during infection with *M. tuberculosis* and probably expand in response to actively multiplying bacilli. Accordingly, subjects with latent *M. tuberculosis* infection had a lower proportion of responding CD4^+^ T_CNP_ cells in the peripheral blood, than tuberculosis patients.

Compared to T_SCM_ cells, T_CNP_ cells express higher levels of CD49d and comparable levels of CXCR3, but they do not express CD95 (19, 20). The α_4_ integrin CD49d associates with β-integrin subunits to form α_4_β_7_ or α_4_β_1_ heterodimers that regulate the trafficking of effector memory T cells to inflamed tissues. Similarly, CXCR3 regulates lymphocyte trafficking in response to its ligands as above reported. Therefore, it is likely that CD4^+^ T_CNP_ cells may be able to rapidly traffic to sites of *M. tuberculosis* infection and engage in the control of bacterial replication by virtue of their ability to secrete the type-1 cytokines, IFN-γ, and TNF-α, which are well known players in anti-mycobacterial protective immune responses (21).

Our findings are reminiscent of the CD8^+^ T cells reported by Pulko et al. (22), that produced IFN-γ but expressed a naive phenotype, and were termed T_MNP_ cells (memory T cells with a naive phenotype). Interestingly, and similar to our findings in tuberculosis, these CD8^+^ T_MNP_ cells were increased in patients with active West Nile virus infection, and their frequency and numbers correlated with the severity of infection.

Thus, our findings demonstrate that CD4^+^ T_CNP_ cells are polyfunctional T cells that differ both phenotypically and functionally from the quiescent CD4^+^ T naive population.

But how are these CD4^+^ T_CNP_ cells (and related subsets) generated ? Sallusto (2) originally proposed a “linear progression” model of differentiation, suggesting that naive T lymphocytes primed by specific antigen hierarchically differentiate into T_CM_ cells which, in turn, further differentiate into T_EM_ cells. Thus, refining and extending this progressive model of T cell differentiation, we now hypothesize that the transition from naive T cells to T_CM_ cells includes the intermediate steps of T_NR_, T_CNP_, T_SCM_ and, possibly, T_MNP_ and other naive-like T cells (Figure 1). This possibility has been demonstrated for T_SCM_ cells and for memory CD8^+^ T_MNP_ cells (6, 22), but needs to be confirmed for all other atypical effector/memory CD4^+^ T cells with a naive-like phenotype.

Our data show that atypical, polarized subsets of naive-like CD4^+^ T cells armed with effector functions can be generated at many points along the differentiation/activation pathway, giving rise to quite heterogeneous effector populations that do not fulfill classical subset phenotypes (23, 24). Besides, we have found that CD4^+^ T_CNP_ cells are not oligoclonal, but show highly heterogeneous Vβ gene segment usage (18). Vβ5.1, Vβ7.1, Vβ8, Vβ12, Vβ13.2, Vβ16, and Vβ17 gene element families were equally expressed among CD4^+^ T_CNP_ and CD4^+^ T_EM_ cells, indicating that the CD4^+^ T_CNP_ cell population might arise in response to similar or even the same *M. tuberculosis* antigens that drove the generation of highly differentiated CD4^+^ T_EM_ cells. Thus, the TCR Vβ data are in agreement with the observation that CD4^+^ T_CNP_ cells compose diverse and likely stable effector/memory populations that have accumulated over time driven, at least in part, by antigen. In fact, if the CD4^+^ T_CNP_ pool would have been derived principally from recently activated cells that were expressing this surface phenotype only transiently, we would have expected some TCR Vβ families overexpressed in the CD4^+^ T_CNP_ population.

However, this does not exclude the possibility that T_CNP_ cells arise by homeostatic proliferation of naive T cells. It has been suggested that CD4^+^ T${}_{\text{N}{\text{R}}^{+}}$ cells do not seem to have arisen by homeostatic proliferation, based on the lack of CD31 expression (5). Conversely, IL-7 and IL-15 have been successfully used to generate T_SCM_ cells (including cytomegalovirus-specific T_SCM_ cells) from naive T cell precursors (19, 25): IL-7 is essential for the development of these cells, whereas IL-15 primarily sustains their expansion (19, 25).

Whether or not the CD4^+^ T_CNP_ cells we have described represent a stable or transient T cell subset (5, 6, 23, 26), if they derive from antigen-driven or cytokine-driven homeostatic proliferation and how this population is related to T_SCM_ (6, 23), T_MNP_ (18), or other populations of effector/memory cells, remain to be determined in future research including studies in experimental animal models.

## Conclusions

Our data support the concept that, despite their seemingly naive cell surface phenotype, the CD4^+^ T_CNP_ cell population contains effector/memory cells, and therefore, the surface markers commonly used to distinguish and purify naive and effector/memory human CD4^+^ T cells, particularly in adults, are inadequate to the latter purpose. The different naive-like effector/memory CD4^+^ T cells reviewed here appear to have important and specific roles in protective immune responses in infectious diseases including tuberculosis.

The question remains whether atypical naive-like effector/memory CD4 T cells should remain incorporated in the naive T cell compartment, since their classical membrane phenotype appears “naïve,” or due to the evidence that these cells are antigen-experienced, they should be considered as an early stage of the effector T cell pool compartment. Our data support the latter view. Further work will be needed to understand whether and how atypical CD4^+^naive-like effector/memory cells can be induced by vaccination, and whether they are associated with long-term memory responses and protection (27). The existence of naive-like T cell with effector functions may also have much wider implications for research in aging, neonatal immunity, and immune-monitoring of the response to vaccination. In both elderly individuals and neonates, immune responses to infections (including tuberculosis) and vaccination are diminished, constituting a major cause of morbidity and mortality. In elderly individuals, a major cause for this impairment is the progressive decline in thymus output with a consequent reduction of the naive T cell pool and expansion of oligoclonal populations of memory cells (28). Alternatively, one could speculate that the altered functional responsiveness of naive T cells in elderly individuals may be the result of a shift from truly naive to more mature naive-like T cells. While our study on T_CNP_ cells (18) did not include old individuals (in fact the oldest patient enrolled in our study was 42-years old), results on other naive-like T cell populations have been inconsistent. Pulko et al. (22) found that both CD8^+^ and CD4^+^ T_MNP_ cell percentages increased in people over 65 years but this increase was relative. In fact, the absolute number of these cells also diminished with age, albeit less rapidly than that of truly naive T cells. In another study, the frequency of circulating T_SCM_ cells did not vary substantially with age (29).

Conversely, the neonatal immune system is largely dominated by truly naive T cells, with very poor representation of memory cells and a relatively high proportion of recent thymic emigrants (RTE) (30). The very poor (if any) memory response in neonates is believed to be compensated in part by the innate-like behavior of RTE due to their capacity to produce IL-8. However, it is necessary to investigate the role that naive-like T cells could play in host defense in early life, with particular attention to understanding and filling the gap in the transition from innate to adaptive responses.

The big question that remains and has not been touched upon is: why would these cells persist in the naive compartment if they have seen antigen, are there any advantages to the cells to remain CD45RA^+^ but CD45RO^−^? Are they only weakly activated? Are they functionally equally powerful as compared to fully differentiated memory T cells? Do they have any unique functional properties? Any thoughts why we would have such atypical cells in such high numbers? A better understanding of the mechanisms involved in the generation and differentiation of the atypical naive-like CD4^+^ T cells may provide novel tools for immunization in early life and in elderly individuals, and more generally for improving vaccination and immunotherapeutic strategies in human infectious disease and cancer.

## Author contributions

All authors listed have made a substantial, direct and intellectual contribution to the work, and approved it for publication.

### Conflict of interest statement

The authors declare that the research was conducted in the absence of any commercial or financial relationships that could be construed as a potential conflict of interest.
